# Soldering of 7075 Aluminum Alloy Using Ni-P and Cu-Cr Electrodeposited Interlayers

**DOI:** 10.3390/ma13184100

**Published:** 2020-09-15

**Authors:** Zbigniew Mirski, Ireneusz Ciepacz, Tomasz Wojdat

**Affiliations:** 1 Department of Metal Forming, Welding and Metrology, Faculty of Mechanical Engineering, Wrocław University of Science and Technology, 50-370 Wrocław, Poland; zbigniew.mirski@pwr.edu.pl; 2 AGA-TEC P.P.H.U., 50-266 Wrocław, Poland; iciepacz@o2.pl

**Keywords:** 7075 aluminum alloy, soldering, electrodeposition, interlayer: Ni-P, Cu-Cr

## Abstract

Direct soldering of the aluminum alloy 7075 is very difficult or even impossible. In order to make it possible, galvanic coatings and the procedures for their application on alloy surfaces were developed. The paper presents structures and mechanical properties of soldered joints of the 7075 alloy, made in indirect way with use of electrolytically deposited Ni-P and Cu-Cr coatings. Application of the newly developed Ni-P and Cu-Cr coatings on base surfaces of the 7075 alloy is described. The results of wettability examination of the S-Sn97Cu3 solder in the droplet test and by spreading on the coatings applied on the 7075 substrates are presented. The wettability angle of both coatings was lower than 30°. The results of metallographic examinations with use of light and electron microscopy are presented. It was shown that adhesion of metallic coatings to the aluminum alloy is good, exceeding shear strength of the S-Sn97Cu3 solder. Shear strength of soldered joint was equal to 35 ± 3 MPa. Measured hardness of the Ni-P interlayer reached high value of 471 HV 0.025.

## 1. Introduction

The 7xxx series aluminum alloys belong to the group of materials with limited bonding ability, especially with use of traditional soldering/brazing methods and arc welding [1,2,3]. Because of their very good mechanical properties, high tensile strength (over 500 MPa) and hardness (over 150 HV) [4,5,6] accompanied by low specific weight, they are more and more often used as construction materials in many industries. This is also favored by a wide range of thermal treatments and quick natural ageing [7,8]. The largest application areas of these alloys are the aircraft and automotive industries [9,10], as well as the space, military and machinery industries [5].

Most of the 7xxx series alloys have the highest strength among all commercial aluminum alloys [3]. Solid solubility of zinc and magnesium in aluminum is variable and their additives make the alloys susceptible to precipitation hardening [3,11]. Moreover, an addition of 1 to 2 wt % of copper increases mechanical properties of the alloys [3]. They can be heat-treated by recrystallization annealing at 390–430 °C or by precipitation hardening composed of supersaturation at 465–480 °C followed by artificial ageing at 120–150 °C [11]. The unfavorable feature of these alloys is their relatively low resistance to corrosion (especially stress corrosion) and to elevated temperature that changes their structure and adversely affects their mechanical properties [11].

During bonding the 7xxx series alloys, significant metallurgical difficulties appear [12]. In particular, traditional welding methods are limited (fusion welding), where the joint is obtained as a result of melting and mixing alloying components of the base materials and the filler metal [13,14]. Mixing the alloying elements often initiates creation of hard and brittle intermetallic phases that adversely affect mechanical properties of joints and, in the case of limited mutual solubility of the alloying elements, can also result in hot cracks in the joints and/or in the heat-affected zone (HAZ) [13]. For this reason, traditional welding methods are replaced by the methods of friction stir welding (FSW), arc braze welding, most often metal inert gas (MIG, TIG) welding with consumable electrodes, and laser braze welding, most often multibeam (trifocal) laser welding [13,14]. Additionally, low-energy methods of welding and braze welding are becoming more popular, e.g., cold metal transfer (CMT) welding [15,16].

**Figure 1:**
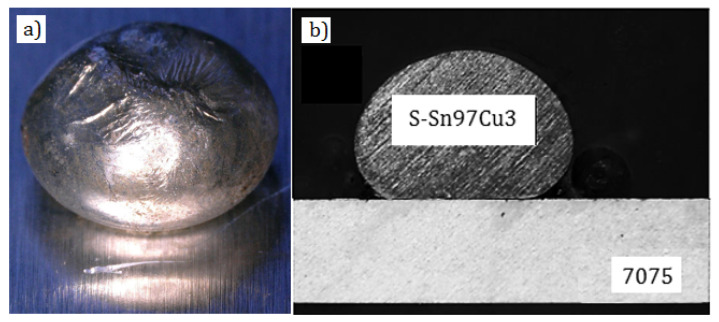
Lack of wettability of S-Sn97Cu3 filler metal on 7075 aluminum alloy substrate: general view (**a**) and cross section (**b**).

The wide range of the liquidus–solidus temperatures from 477 to 635 °C for the 7075 alloy [5] precludes its brazing with use of traditional Al-Si-based solders. With use of silumin solders, the soldering process must be run at ca. 600 °C [17,18]. It is also impossible to join the 7xxx series alloys by soldering directly, because of a shortage of filler metals and, first of all, fluxes ensuring good wettability (Figure 1) and, in consequence, creation of high-quality soldered joints.

In practice, the soldering of high melting alloys, non-wettable by solders, is often carried-out in an indirect way. This consists in creating intermediate layers (coatings), mostly metallic and well wettable by the solders, on surfaces of the materials to be joined. In this way, for example, aluminum with copper [19], graphite with copper [20] or aluminum [21] and ceramics with metals [22] are soldered. Coatings can be applied on surfaces of the base materials in different ways, starting from galvanic methods, through the chemical (CVD) and physical vapor deposition (PVD) methods, to the methods of thermal plasma spraying or low- and high-pressure cold gas spraying.

Within the research, trials were undertaken to solder the 7075 aluminum alloy with use of the electrolytically deposited Ni-P and Cu-Cr coatings, newly developed by the authors [23,24]. With regard to the wide liquidus–solidus range of the 7075 alloy, a tin-based solder was used for soldering. This restricts the potential application area to the joints not carrying high service loads, but maintaining metallic continuity and ensuring good electric or thermal conductivity.

## 2. Materials and Methodology

Within the research, specimens of the 7075-T6 alloy were joined in indirect way, with use of Ni-P and Cu-Cr layers. The 7075 aluminum alloy is characterized by high mechanical strength (tensile strength is 480–540 MPa), but it has relatively low resistance to corrosion, especially stress corrosion. Due to the high sensitivity to high temperatures, it is preferable to bond it at low temperatures, using the soldering technology. For this reason, the technologies of furnace and flame soldering were applied. As a solder, the S-Sn97Cu3 alloy was used, with melting point between 232 and 290 °C [25]. A flux based on zinc chloride and ammonium chloride was also used, recommended for soldering copper and copper alloys and nickel with use of tin-based solders. The flux remains active up to 316 °C [25]. Chemical compositions of base metal and filler metal according to [26,27] as well as results of the performed spectral analysis are shown in Table 1.

For wettability examination of the solder in the droplet test and by the spreading method, specimens of the 7075 alloy 30 × 30 mm and 3 mm thick were used. For the soldered lap joints, specimens 25 × 80 mm and 3 mm thick were used.

After mechanical grinding and chemical cleaning, metallic coatings were electrolitycally deposited on the specimens. Electrolysis was carried-out in two different, newly developed galvanic baths, with the parameters selected to obtain the Ni-P and Cu-Cr coatings about 12 mm thick. Such thickness of the coatings should prevent their brittleness, but ensure their tightness and durability during soldering. The thickness and the chemical composition of the applied coatings were measured using an X-Ray fluorescence analyser FischerScope XRAY XDL-B type X-ray from FISCHER GmbH (Achern, Badenia-Wirtembergia, Germany).

After applying the coatings, their wettability was assessed by measurements of surface free energy (SFE) and its polar and dispersive components with use of an analyser Krüss DSA HT 1200 (Krüss GmbH, Hamburg, Germany), integrated with the computer program DSA3. As reference liquids, distilled water, diiodomethane and ethylene glycol with known surface energies and known polar and dispersive components were applied, see Table 2 [28]. Single droplets of each liquid were placed on properly prepared surfaces of the examined specimens and the wetting angle *θ* was read from the program with accuracy to 0.1°. All the *θ* values were determined by the Owens–Wendt–Rabel–Kaelble (OWRK) method [29]. In this method, the wettability and adhesion depend on the influence of disperse and polar interactions of the measuring liquid. Using the OWRK method, it is possible to determine and optimize the influence of various processing methods (e.g., plasma treatment or coating) on the value of adhesive interactions in processes, such as bonding, painting, hydrophobic coatings, etc. using the effect of changing the polarization during contact between surfaces of different polarity [28,29].

The soldering properties were definitely determined in the wettability test, carried-out by spreading the S-Sn97Cu3 solder on the 7075 substrates with applied coatings. Weighted 0.1 g doses of the solder with a half this weight of flux were placed on the samples. Next, the samples (5 for each series) were placed on a ceramic support and together inserted to the furnace heated to 300 °C. The electric furnace was Czylok FCF 7SM 2.6 kW (Czylok, Jastrzębie-Zdrój, Śląsk, Poland) with operating temperature to 1100 °C. Because of relatively high thermal inertia of the samples, the flux was activated and the solder started melting after 120 s. From that moment, the samples were kept in the furnace for another 30 s. The samples taken out from the furnace were cleaned of residues of slag and subjected to further examinations.

The joints for metallographic examinations and mechanical testing, i.e., for static tensile shear test and Vickers hardness measurements, were prepared by flame soldering. Because of low process temperature, heating with propane-air flame was used. In order to obtain repeatable results, distance elements in the form of 0.2 mm diameter steel wires were used, guaranteeing constant width of the soldering gap. The lap was 10 mm wide. From the moment the solder started melting, the joints were heated for a further 5 s and then cooled in air. The joints for metallographic examinations were cut in half, mounted in resin, ground and polished to obtain microscopic samples. Tensile shear strength was carried out on a universal mechanical testing machine—Zwick/Roell ZMARTPRO (Zwick-Roell GmbH, Badenia-Wirtembergia, Ulm, Germany). The Vickers hardness measurements were carried out on the cross-sections of the soldered joints using a low indenter load of 25 G. The hardnesses were measured using a Sinowon PMT3 testing machine (Sinowon, DongGuan, Guangdong, China).

## 3. Results and Discussion

### 3.1. Electrodeposition of Ni-P and Cu-Cr Coatings

Conditions of the coating deposition were determined by means of the Pourbaix diagram [30]. It results from the diagram that the 7075 alloy shows a significantly limited area of corrosion resistance in the range of possible galvanic baths. So, even if the 7075 alloy could be coated in highly acidic baths, it is practically impossible because of the position of aluminum in the galvanic series. Therefore, coating of the alloy was carried-out with use of intermediate layers, strongly adhered to the substrate.

For deposition of galvanic coatings, a laboratory station was built, composed of a power supply (Elektro-Tech type ETZ 10/10, Elektrotech, Kryniczno, Dolny Śląsk, Poland) with steplessly-adjusted amperage from 0 to 10 A and voltage from 0 to 10 V, a magnetic stirrer (IKA type ETS 06, IKA Sp. z o.o., Warsaw, Mazowieckie, Poland) with steplessly adjusted rotational speed and a heating system with temperature control. On the stirrer, a 1 dm^3^ beaker was placed, containing the electrolytic bath and the anode 50 × 120 × 5 mm. The anode used for depositing the Cu-Cr coating was made of rhodium-coated titanium and that for depositing the Ni-P coating—of cathodic nickel.

The deposition process was preceded by proper preparation of the substrate surface. At the first stage, the samples were ground with abrasive papers No. 150 and 280, and next subjected to washing in 5% SurTec 131 solution (Surtec Poland Sp. z o.o., Janikowo, Wielkopolska, Poland) at 40 °C for 5 min. Then, the samples were rinsed under tap water and decapped in 5% SurTec 495L solution (Surtec Poland Sp. z o.o., Janikowo, Wielkopolska, Poland) at 30 °C for 3 min. Before applying the due coatings, intermediate layers were deposited. This process was preceded by a treatment with low-temperature argon plasma for 30 s, in order to increase adherence of the coatings to the aluminum substrate. A favorable effect of low-temperature plasma treatment on adherence of copper coating to a substrate of graphite composite was indicated in [31]. Preliminary researches also showed that low-temperature plasma treatment resulted in an over 40% increase of the force required to scratch the Cu-Cr coating applied on the aluminum substrate.

First, the specimens were chemically zinc plated in SurTec 652Q bath Surtec Poland Sp. z o.o., Janikowo, Wielkopolska, Poland at 15 to 40 °C for 1 min. Next, preliminary electrolytic copper plating in SurTec 864 bath Surtec Poland Sp. z o.o., Janikowo, Wielkopolska, Poland with pH 9.5 was carried-out at 55 °C for 2 min. The cathode current density was 0.5 A/dm^2^ and the anode was made of oxygen-free copper (OFHC). On this way prepared substrates, the due coatings designed for soldering were applied. To that end, two baths were developed:

- Electroplating Cu-Cr bath—weakly acidic bath for depositing a copper-chromium layer containing 0.9 to 1.2 wt % Cr [23];
- Electroplating Ni-P bath—new acidic bath for depositing a nickel-phosphorus layer containing 12 wt % P [24].

The Cu-Cr alloy can be used as a coating with increased abrasion resistance. The related references do not mention electroplating baths for depositing the alloyed Cu-Cr coatings, but the information can be found about obtaining these coatings in metallurgical processes, mostly applied in power industry.

The compositions of the bath and process parameters of depositing the Cu-Cr coating are the following:

- 8–12 g/dm^3^ of chromium metal in form of chromium chloride III;
- 12–15 g/dm^3^ of copper metal in form of copper chloride II;
- 80–120 g/dm^3^ of ammonium chloride as the conducting salt;
- pH of the solution within 3.8 to 4.5;
- process temperature within 55 to 65 °C;
- cathodic current density from 1.5 to 3.0 A/dm^2^;
- process time from 40 to 60 min.

The Ni-P alloy is applied, among others, in manufacture of filler metals used for flux-less brazing. It can be used in electroplating technique as a decorative nickel coating with increased corrosion resistance (an alternative for the chromium coating).

The chemically applied Ni-P coatings are used as technical coatings with high corrosion resistance that depends on concentration of phosphorus. Chemical coating with nickel is also applied in the plastics industry.

In the literature, publications can be found concerning developed baths for electrochemical deposition of the Ni-P coating. Baths for deposition of such a coating containing 18 wt % P, being an alternative for a chromium coating, are developed by German and Italian companies [32]. In these baths, phosphorus ions are delivered by the sodium salt of phosphoric acid III, added at 5–7 wt %. The coatings are applied as decorative coatings in manufacture of fixtures and fittings, parts of household appliances and in the automotive industry.

The compositions of the bath and process parameters of depositing the developed Ni-P coating are the following:

- 15–18 g/dm^3^ of nickel metal in form of nickel sulphate II;
- 100–150 g/dm^3^ of phosphorus in form of nitrilotris(methylene)phosphonic acid;
- 100 g/dm^3^ of citric acid;
- pH of the solution within 1.5 to 2.5;
- process temperature within 45 to 55 °C;
- cathodic current density from 1.0 to 2.5 A/dm^2^;
- process time from 15 to 25 min.

### 3.2. Evaluation of Applied Coatings

The electrolytically applied coatings were subjected to a preliminary analysis. Their thickness and chemical composition was measured with an X-Ray fluorescence analyzer FISCHERSCOPE X-RAY XDL-B made by Fischer GmbH (Achern, Badenia-Wirtembergia, Germany). Concentration of Cr (wt %) was measured for the Cu-Cr coating, concentration of P (wt %) was measured for the Ni-P coating and the balances were respectively Cu and Ni contents. The measurement applications for the Ni-P coating are commercial products, but the application for measuring the Cu-Cr coating was developed by the company Helmut Fischer GmbH Achern, Badenia-Wirtembergia, Germany for the needs of this research.

Thickness measurements and chemical compositions of the coatings applied on the 7075 substrates are shown in Table 3.

Adhesion testing of the coatings was carried-out according to EN ISO 2819:2018 [33]. Preliminary measurements were taken by the “thermal shock” method and, after a positive result, adhesion was determined by the scratching method, with use of the Micro-Combi-Tester made by CSM Instruments (Needham Heights, MA, USA). The tester determines the surface profile of both the substrate and the coating. During the peeling process, force and acoustic signal are recorded. Moreover, the surface profilogram is recorded, as well as penetration depth in the coating and in the substrate.

After electrolysis, all samples were kept in an electric oven at 200 °C for ca. 30 min and then dropped to a container with water at ambient temperature. After 1 min, peeling of the coating was assessed visually. All the samples positively passed the thermal shock test with no visible peeling.

Adhesion testing with the Micro-Combi-Tester was started from measurements of scratching force of the uncoated substrate and next, the force required to peel the coating from the coated substrate was measured. The test was carried-out on the distance 5 mm long, at pressure force 29 N. The coating was peeled at the same time in two places and average value of the used forces was reported.

**Figure 2:**
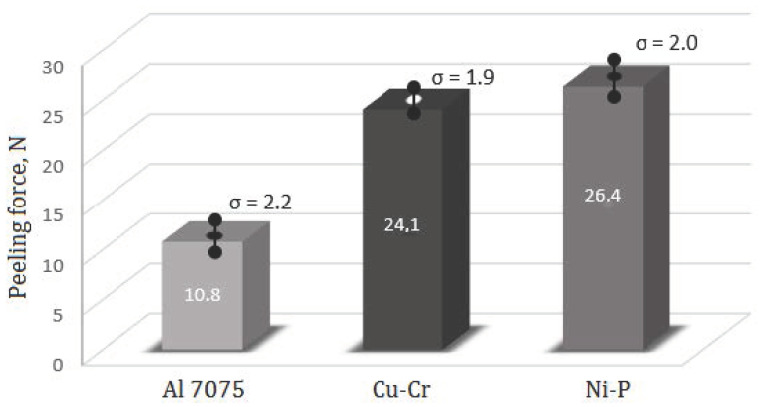
Results of the scratch test.

The adhesion test was carried-out as follows. The peeling head loaded with the above-mentioned force started peeling-off the coating and the computer recorded all the events accompanying the process (e.g., rupture of the coating). After penetrating the substrate material, microscopic photographs were taken in all the points where the measurement continuity was disturbed and finally the entire scratching was photographed. After the test, a print-out is obtained, containing the surface profilogram, size of the force required to peel-off the coating, sizes of the forces occurring in the disturbances and the complete photographic documentation. Sizes of the forces required to scratch the substrate with no coating and the substrates with electrolytically deposited coatings are shown in Figure 2. The results are averages of 10 measurements.

**Figure 3:**
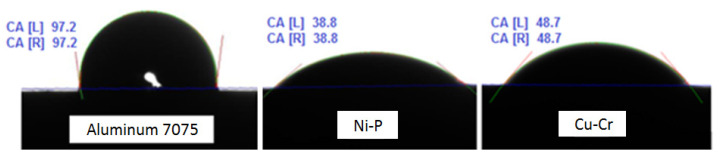
Wettability of the substrate and the coatings with distilled water.

Wettability of the 7075 substrate and of the applied Cu-Cr and Ni-P coatings was preliminarily determined by the droplet test, as described above. The exemplary wettability of the 7075 surface and the coatings with distilled water is shown in Figure 3. It can be seen that the wettability of the coatings is significantly better than that of the base metal.

Average values of wetting angles for various reference liquids are given in Table 4. Additionally presented are the values of surface free energy and its dispersive and polar components. Surface energy of the applied coatings is higher than that of the substrate. Higher surface energy is related to lower surface tension and thus better wettability (smaller wetting angles with individual reference liquids).

### 3.3. Wettability Test

As was mentioned above and shown in Figure 1, the 7075 alloy substrate is not wettable by soft solders. As a result, it is impossible to make soldered joints directly. Usefulness of the applied Cu-Cr and Ni-P coatings for soldering was determined by measuring wettability of their surfaces by the tin-based solder. Samples were made in the way described above. According to the wettability criteria, the smaller wettability angle and the larger surface areas of the spread droplets, the better are soldering properties of the substrate. It is accepted that good wettability occurs, when the wetting angle is below 30° and tends to 0° [34,35,36].

**Figure 4:**
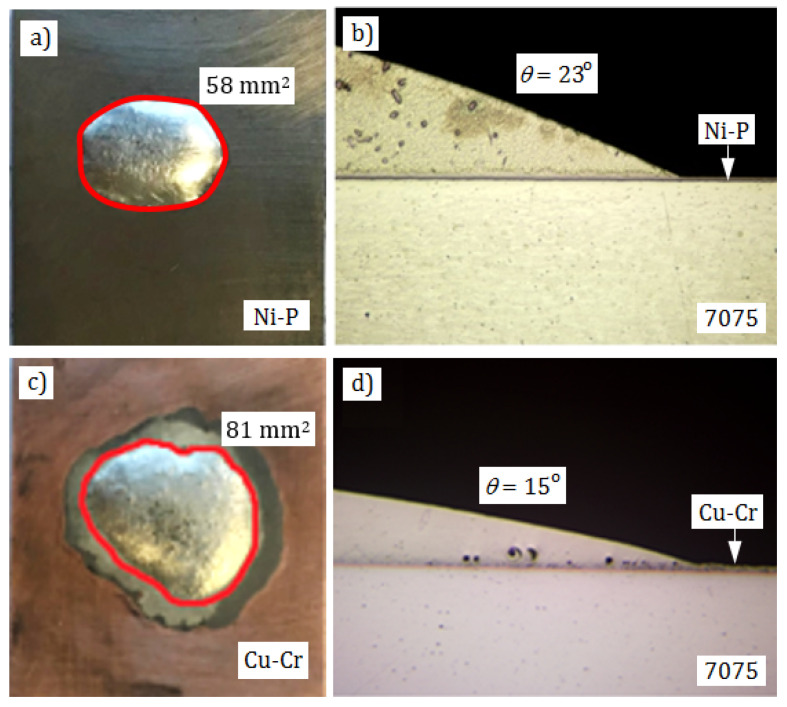
Spreadability and wettability of S-Sn97Cu3 solder on Ni-P (**a**,**b**) and Cu-Cr (**c**,**d**) coatings.

Mean size of planimetered surfaces on that 0.1 g of the solder spread was 74 mm^2^ (*σ* = 7.2 mm^2^) for the Cu-Cr coating and 59 mm^2^ (*σ* = 8.1 mm^2^) for the Ni-P coating. Exemplary spreadability areas of solders and cross-sections of solder droplets on the substrates used for determination of wettability angles are shown in Figure 4. Mean wettability angle values were 28° (*σ* = 7.3°) for the Cu-Cr coating and 17° (*σ* = 4.8°) for the Ni-P coating. According to the evaluation criteria [34,35,36], such wettability angle values are indicative of good wettability of coatings and should ensure good conditions for making good-quality soldered joints. In the case of the Cu-Cr coating, a change of color can be seen within the flux action area (Figure 4c), but continuity of the coating was not broken.

### 3.4. Metallographic Evaluation of Soldered Joints

**Figure 5:**
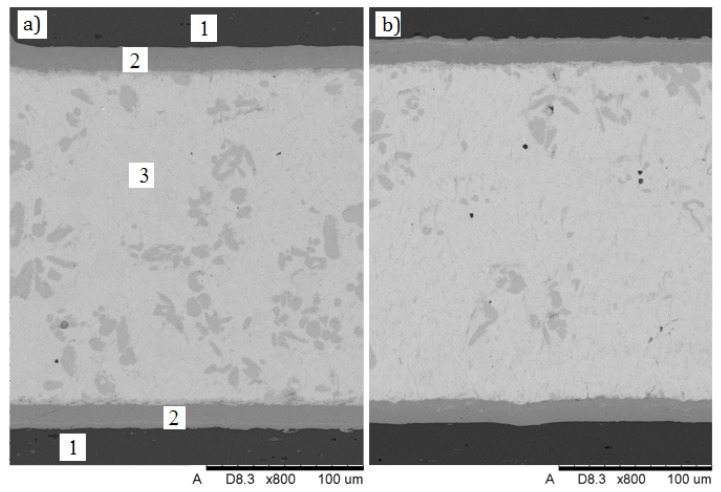
Microstructure of soldered joint made via Cu-Cr interlayer, two different part of joint (**a**,**b**): 1—substrate (aluminum 7075), 2—Cu-Cr interlayer, 3—solder.

As mentioned before, lap joints with 10 mm long laps and constant width of soldering gaps fixed with distance elements dia. 0.2 mm were prepared for metallographic examinations and mechanical testing. The joints were made by flame soldering, using a propane-air burner. Except for very tiny gas pores and residues of flux, no other soldering imperfections were found. After soldering, the coatings were still continuous and well adhered to the 7075 alloy substrate. The joint made with the intermediate 12 µm thick Cu-Cr layer is shown in Figure 5. Figure 5a,b shows different sections of soldered joints. Both microstructures are very similar, but in Figure 5b a trace amount of very fine gas pores are visible. It results from the Cu-Sn equilibrium system [37] and the EDS (Energy Dispersive Spectroscopy) analysis, that microstructure of the solder layer consists of eutectic mixture Sn + Cu_6_Sn_5_ with grey primary crystals of the solid solution Cu_6_Sn_5_. The Cu-Cr coating adheres well to the aluminum 7075 substrate. There are no visible solder incompatibilities lowering the quality of soldered joint, except very small gas pores or flux residue.

**Figure 6:**
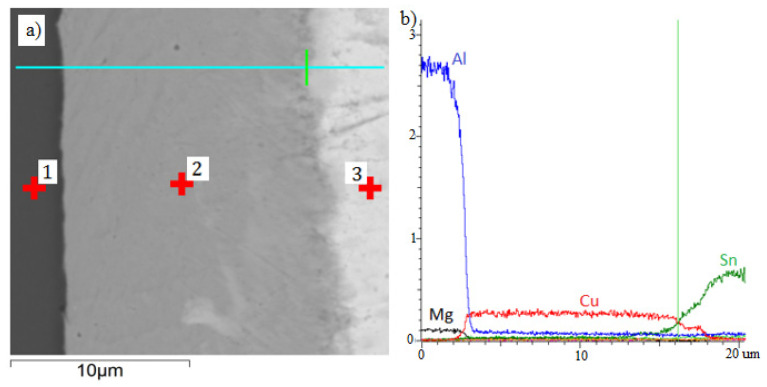
BSE (Back Scattered Electrons) image of transition zone (**a**) and linear EDS analysis of soldered joint made via Cu-Cr interlayer (**b**): 1—substrate (aluminum 7075), 2—Cu-Cr interlayer, 3—solder.

Linear distribution of elements in the joint is characteristic for the analyzed system (Figure 6). The coating (2) is composed of copper (98.99 wt % Cu) and chromium (1.01 wt % Cr), which is in accordance with specification of the electroplating bath. The solder (3) is composed of tin (96.7 wt % Sn) and copper (3.3 wt % Cu). Because of low temperature of soldering, no diffusion zones are visible in the joint. During the soldering process, the elements in the coating do not moving into the solder or back from the solder to the coating.

**Figure 7:**
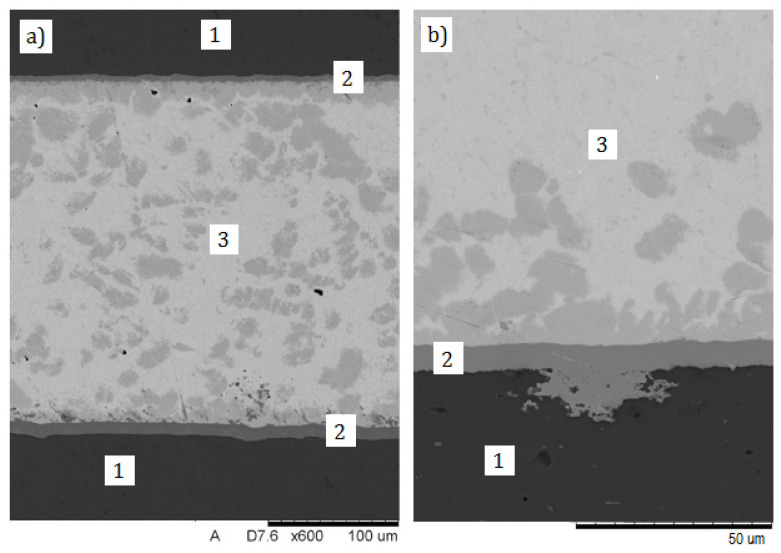
Microstructure of soldered joint made via Ni-P interlayer (**a**), filled surface defects of the substrate (**b**): 1—substrate (aluminum 7075), 2—Ni-P interlayer, 3—solder.

In the joint soldered via the Ni-P interlayer, no significant soldering imperfections of microstructure were found, either. The coating is well adherent to the 7075 substrate on the entire length of the joint (Figure 7a). The Ni-P coating well filled surface defects of the substrate (Figure 7b), creating strong mechanical anchor points. As before, microstructure of the solder layer consists of eutectic mixture Sn + Cu_6_Sn_5_ with grey primary crystals of the solid solution Cu_6_Sn_5_.

The morphology of both solder in soldered joints made with use Cu-Cr (Figure 5) and Ni-P interlayer (Figure 7) are very similar. Shape of grey primary crystals of the solid solution Cu_6_Sn_5_ in both soldered joint are similar, but more of crystals are in the joint made with Ni-P. The difference may most likely result from the difference in soldering time of both joints. The joints were made by manual flame soldering, where precise control of the soldering time is difficult.

**Figure 8:**
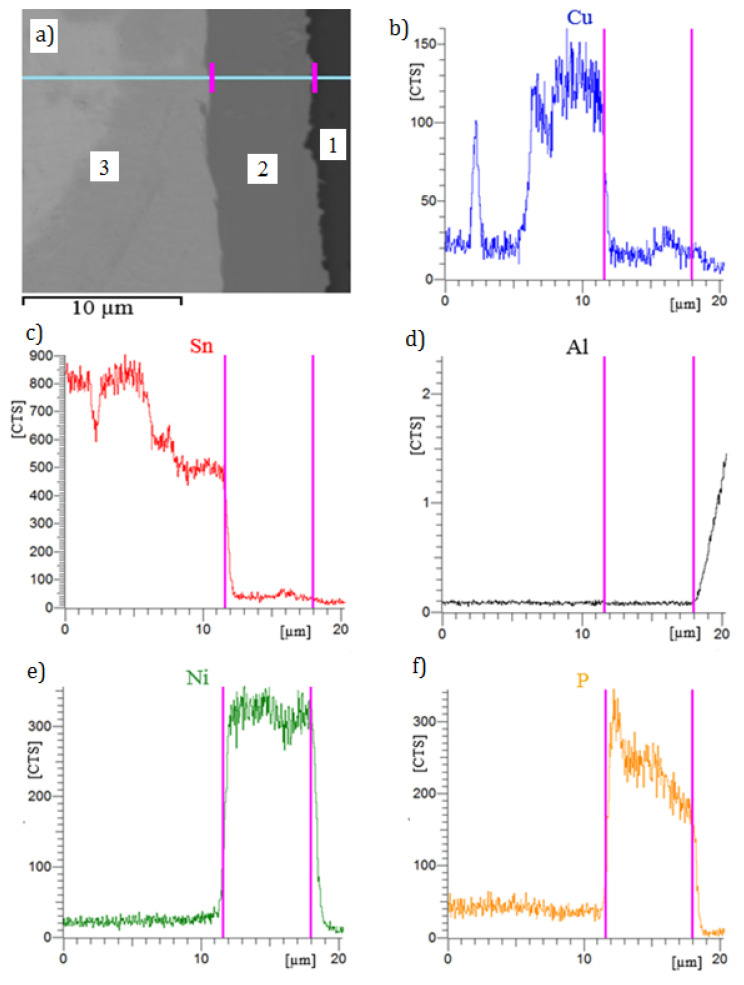
BSE (Back Scattered Electrons) image of transition zone of soldered joint made via Ni-P interlayer (**a**) and linear content of elements (**b**–**f**): 1—substrate (aluminum 7075), 2—Ni-P interlayer, 3—solder.

The place selected in the joint structure for the EDS analysis is shown in Figure 8a. In Figure 8b–f, the vertical lines mark the Ni-P coating and the linear distribution of elements in individual zones of the joint. In this joint, no diffusion zones or elements moving from the solder to the coating or back from the coating to the solder were found, either. The coating Ni-P (2) is composed of nickel (87.7 wt % Ni) and phosphorous (12.3 wt % P), which is in accordance with specification of the electroplating bath. Concentration of phosphorus in the coating increases with the distance from the aluminum 7075 substrate (Figure 8f) from 11.5 to 13.2 wt %. This is a regularity resulting from the electrolysis course. The solder (3) is composed of tin (96.9 wt % Sn) and copper (3.1 wt % Cu). No diffusion mechanisms were found due to the low temperature of the soldering process.

As opposed to the coatings applied by the low-pressure cold spray (LPCS) method, those deposited electrolytically are not porous, which is favorable in their application to soldering. This results from the fact that porosity of the coatings applied by the thermal spray methods is conducive to creation of gas pores in the soldered joint [38].

### 3.5. Tests of Mechanical Properties of Soldered Joints

Tensile shear tests of the joints made with use of the Cu-Cr and Ni-P interlayers were carried-out on a universal testing machine Zwick/Roell Zmart-PRO (Zwick-Roell GmbH, Badenia-Wirtembergia, Ulm, Germany). The soldered joints were positioned in the machine grips with use of suitable distance inserts and then stretched at 2 mm/min. Five sets of soldered joints were prepared for each coating. Before the shear test, flashes of solder were mechanically removed on both sides of the joint. Results of static tensile shear test of the soldered joints are shown in Table 5.

**Figure 9:**
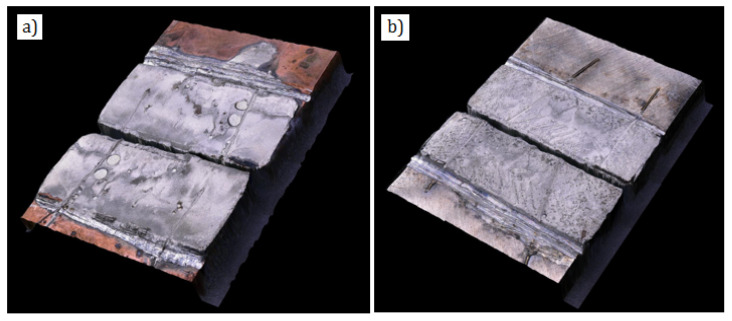
Cohesive fractures within solder after shear strength test: joints soldered with Cu-Cr (**a**) and Ni-P (**b**) interlayer.

Shear strength of the soldered joints was in both cases similar at ca. 35 MPa. The destruction mechanism of both joints was of cohesive nature and occurred within the solder layer (Figure 9). What is important, the Cu-Cr and Ni-P coatings maintained their adherence with the substrate. So, it can be supposed that the joints can withstand higher loads, if mechanical properties of the used solder are higher. Strength of the joints with electrolytically deposited coatings is over 40% higher than that of the joints with the coatings sprayed by the LPCS method [38], where destruction occurred within the coatings as a result of their decohesion. As reported in [38], the reason could be high porosity of the LPCS sprayed coatings.

**Figure 10:**
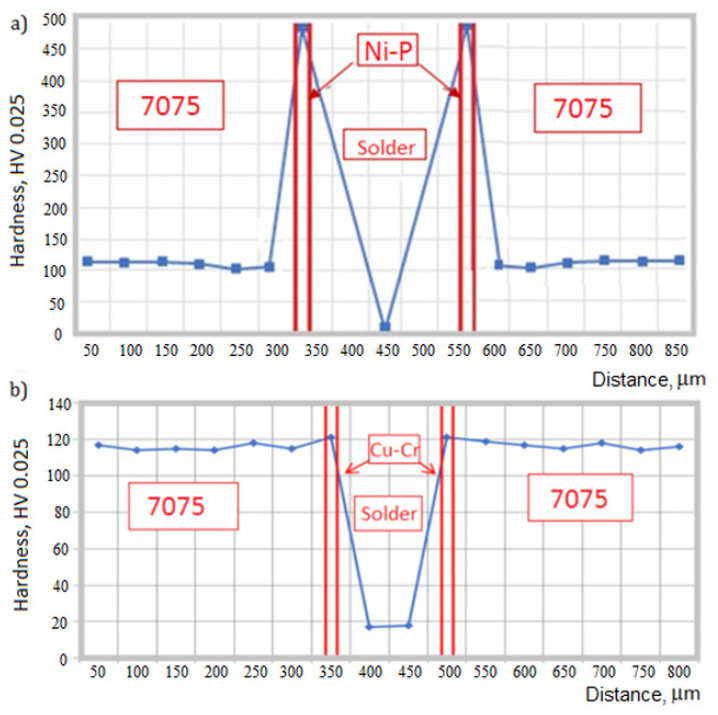
Distribution of hardness HV 0.025 in soldered joints made via Ni-P (**a**) and Cu-Cr (**b**) interlayers.

Vickers hardness was also measured in individual zones of the soldered joints [39]. Due to the small thickness of the electrolytic layers, the load of the penetrator was 25 G. Distribution of hardness in the joints is shown in Figure 10. The presented points are average values of 10 measurements.

Hardness of the Ni-P coating, being on the average 471 HV 0.025 (*σ* = 14.4 HV 0.025), is much higher than that of the substrate metal. As reported in [40], hardness of electrolytically deposited Ni-P coatings containing 16 wt % P is ca. 600 HV. The coatings with higher hardness over 700 HV can be obtained by additions of ceramic particles SiC or B_4_C [40]. Hardness of the Cu-Cr coating is almost the same as that of the metal substrate and equals on the average to 121 HV 0.025 (*σ* = 8.7 HV 0.025). It was indicated in [41] that hardness of the Cu coating applied electrolytically on the Cr layer previously applied on the carbon-steel substrate ranges from 42 to 84 HV and depends on voltage used during electrodeposition. The lowest hardness in the soldered joints shows the S-Sn97Cu3, on the average 18.9 HV 0.025 (*σ* = 3.6 HV 0.025).

## 4. Conclusions

The newly developed, electrolytically deposited Ni-P and Cu-Cr coatings do well in soldering the hardly solderable aluminum alloy 7075. They show high surface energy, so they are readily wettable by the reference liquids and solders. They are characterized by good solderability demonstrated by the wetting angle values below 30°. The coatings are well adhesively bonded with the substrate and do not become disbonded during soldering. With these coatings, it is possible to make soldered joints with shear strength ca. 35 MPa, determined by shear strength of the used tin-based solder. Hardness of the coatings is relatively high, equal to 121 HV 0.025 for the Cu-Cr coating and even to 471 HV 0.025 for the Ni-P coating. In addition to intermediate layers in soldering, these coatings can be also used as decorative coatings or perform other technical functions, like protection against corrosion or abrasive wear.

## Figures and Tables

**Table 1:** Chemical composition of base metal and filler metal.

Chemical Composition, wt %									
Element	Si	Fe	Cu	Mn	Mg	Cr	Zn	Ti	Al
Base Metal 7075 alloy	Max. 0.40	Max. 0.50	1.20–2.00	Max. 0.30	2.10–2.90	0.18–0.28	5.10–6.10	Max. 0.20	Rem.
SP Analysis *	0.12	0.07	1.80	0.09	2.88	0.19	6.04	0.06	Rem.
Element	Cu	Fe	In	Bi	Pb	Ag	Ni	Other	Sn
S-Sn97Cu3	2.50–3.50	Max. 0.02	Max. 0.10	Max. 0.07	Max. 0.10	Max. 0.10	Max. 0.01	Max. 0.186	Rem.
SP Analysis *	2.65	0.01	0.08	0.04	0.05	0.09	0.01	0.18	Rem.

* SP Analysis—Spectral Analysis.

**Table 2:** Properties of selected measuring liquids.

Liquid	Surface Free Energy (SFE), *γ_w_* [mJ/m^2^]	Dispersion Component, *γ_wD_* [mJ/m^2^]	Polar Component, *γ_wP_* [mJ/m^2^]
Distilled Water	72.8	21.8	51.0
Diiodomethane	50.8	48.5	2.3
Ethylene Glycol	48.0	29.0	19.0

**Table 3:** Thickness and composition of the deposited coatings.

Coating	Thickness, µm	Average Thickness, µm	Element Content, wt %			
			Cr	AV Cr	P	AV P
Cu-Cr	12.9	12.3 (*σ* = 0.9)	1.17	1.12 (*σ* = 0.09)	_	_
	13.0		1.17			
	12.9		1.08			
	11.9		1.19			
	10.9		0.98			
Ni-P	12.7	12.1 (*σ* = 0.7)	_	_	12.2	11.6 (*σ* = 0.5)
	12.8				11.2	
	11.9				11.9	
	11.0				10.9	
	11.9				11.9	

*σ*—standard deviation, AV—average.

**Table 4:** Surface free energy and wettability of electrodeposited coatings and 7075 aluminum alloy substrates.

Substrate	Surface Free Wnergy (SFE), *γ_w_* [mL/m^2^]	Dispersion Component, *γ_wD_* [mJ/m^2^]	Polar Component, *γ_wP_* [mJ/m^2^]	Contact Angle, °		
				Distilled Water	Diiodomethane	Ethylene Glycol
7075	41.7	36.8	4.9	92.1	68.2	57.9
Cu-Cr	58.8	42.5	16.3	49.4	43.8	41.2
Ni-P	66.9	43.1	23.8	38.7	32.4	22.6

**Table 5:** Shear strength of soldered joints made via Cu-Cr and Ni-P interlayers.

No.	Lap Joint		Shear Force *F_t_* [N]	Shear Strength *R_t_* [MPa]	Average Shear Strength *R_ta_* [MPa]	Fracture Type
	Dimensions [mm × mm]	Area of Joint [mm^2^]				
Cu-Cr Interlayer						
1	24.0 × 9.8	235	8600	36.6	35.4 (*σ* = 1.2)	Cohesive
2	24.0 × 10.0	240	8100	33.8		
3	23.8 × 9.7	231	8500	36.3		
4	23.9 × 9.7	232	8300	35.8		
5	23.4 × 9.9	232	8250	34.5		
Ni-P Interlayer						
1	25.1 × 9.1	228	7550	33.1	33.2 (*σ* = 0.8)	Cohesive
2	25.1 × 8.3	208	6700	32.2		
3	25.0 × 8.5	212	7300	34.4		
4	25.3 × 8.8	223	8200	32.8		
5	25.2 × 8.6	218	7950	33.5		
